# *Ehrlichia* effector SLiM-icry: Artifice of cellular subversion

**DOI:** 10.3389/fcimb.2023.1150758

**Published:** 2023-03-07

**Authors:** Nicholas A. Pittner, Regina N. Solomon, Duc-Cuong Bui, Jere W. McBride

**Affiliations:** ^1^ Department of Pathology, University of Texas Medical Branch, Galveston, TX, United States; ^2^ Department of Microbiology and Immunology, University of Texas Medical Branch, Galveston, TX, United States; ^3^ Center for Biodefense and Emerging Infectious Diseases, University of Texas Medical Branch, Galveston, TX, United States; ^4^ Sealy Institute for Vaccine Sciences, University of Texas Medical Branch, Galveston, TX, United States; ^5^ Institute for Human Infections and Immunity, University of Texas Medical Branch, Galveston, TX, United States

**Keywords:** *Ehrlichia*, tandem repeat protein, short linear motif, effector, Notch, Wnt, Hedgehog, nucleomodulin

## Abstract

As an obligately intracellular bacterial pathogen that selectively infects the mononuclear phagocyte, *Ehrlichia chaffeensis* has evolved sophisticated mechanisms to subvert innate immune defenses. While the bacterium accomplishes this through a variety of mechanisms, a rapidly expanding body of evidence has revealed that *E. chaffeensis* has evolved survival strategies that are directed by the versatile, intrinsically disordered, 120 kDa tandem repeat protein (TRP120) effector. *E. chaffeensis* establishes infection by manipulating multiple evolutionarily conserved cellular signaling pathways through effector-host interactions to subvert innate immune defenses. TRP120 activates these pathways using multiple functionally distinct, repetitive, eukaryote-mimicking short linear motifs (SLiMs) located within the tandem repeat domain that have evolved *in nihilo*. Functionally, the best characterized TRP120 SLiMs mimic eukaryotic ligands (SLiM-icry) to engage pathway-specific host receptors and activate cellular signaling, thereby repurposing these pathways to promote infection. Moreover, *E. chaffeensis* TRP120 contains SLiMs that are targets of post-translational modifications such as SUMOylation in addition to many other validated SLiMs that are curated in the eukaryotic linear motif (ELM) database. This review will explore the extracellular and intracellular roles TRP120 SLiM-icry plays during infection - mediated through a variety of SLiMs - that enable *E. chaffeensis* to subvert mononuclear phagocyte innate defenses.

## Introduction

In recent years, the obligately intracellular pathogen *Ehrlichia chaffeensis* has become recognized for its profound ability to manipulate host cell signaling (Byerly et al., 2021). *E. chaffeensis* causes human monocytic ehrlichiosis (HME), a tick borne zoonosis that can have life-threatening manifestations including acute respiratory distress, meningitis and multisystem failure (Patel and Byrd, 1999). *E. chaffeensis* is an alpha-proteobacterium (family Anaplasmataceae, order Rickettsiales) (Dumler et al., 2001) that infects mononuclear phagocytes (Paddock and Childs, 2003). The family Anaplasmataceae includes genera *Ehrlichia, Anaplasma*, *Neorickettsia*, and *Wolbachia*, which comprise a group of obligately intracellular bacteria, many of which are emerging zoonotic human pathogens of public health importance.

Infection of the mononuclear phagocyte by *E. chaffeensis* is accomplished by successful evasion of innate immune defenses which is achieved in part by effector proteins secreted *via* membrane bound secretion systems. Type I and type IV secretion systems (T1SS and T4SS) have been identified and functionally confirmed (Dunning Hotopp et al., 2006; McBride et al., 2011; Liu et al., 2012; Yan et al., 2018), and several effectors of these secretion systems that play a role in infection have been identified (Kumagai et al., 2010; Liu et al., 2012; Lina et al., 2016).

The T1SS is an ATP-binding cassette (ABC) transporter that allows for the secretion of effector proteins in a one-step process. The ehrlichial genome encodes three T1SS components: HylB, the inner membrane ATP-binding cassette protein (ECH0383), HylD, the membrane fusion protein (ECH0970), and TolC, the outer membrane protein (ECH1020) that form the secretion nano-machine (Delepelaire, 2004). Within the bacterial cytoplasm, unfolded proteins with a C-terminal type I secretion signal sequence are recognized by HylB and translocated across the inner membrane in an ATP-dependent manner. Bacterial substrates pass through HylD, a homodimeric pore that spans the periplasm, where it interacts with TolC within the outer membrane and is released to the cytoplasm of the host cell (Delepelaire, 2004; Spitz et al., 2019). *E. chaffeensis* utilizes the T1SS to secrete multiple tandem repeat effector proteins into the host cell.

*Ehrlichia* tandem repeat protein (TRP) T1SS effectors share similarities with the repeats-in-toxins (RTX) family of proteins such as exotoxins, lipases, and adhesins, including unique glycine- and aspartate-rich tandem repeats, ATP transporter homology, and non-cleavable C-terminal T1SS signals (Wakeel et al., 2011). TRP effectors are highly immunoreactive, eliciting vigorous host antibody responses directed at linear antibody epitopes (Byerly et al., 2021). Investigations have revealed that TRPs interact with numerous host proteins involved in cell signaling and immune response, cytoskeletal organization, post-translational modifications (PTMs), transcriptional and translational regulation, intracellular trafficking and apoptosis (Byerly et al., 2021). Specifically, TRP32 interacts with a diverse group of host cell targets that influence intracellular survival (Luo and McBride, 2012). TRP47 enters the nucleus *via* a MYND-binding domain-dependent mechanism and predominantly binds enhancers of host genes associated with signal transduction, cytoskeletal organization, and immune response (Kibler et al., 2018). TRP75 has been shown to interact with host proteins involved in homeostasis, cytoskeleton organization, and apoptosis regulation to promote infection (Luo et al., 2018). Notably, TRP120 is the most characterized *E. chaffeensis* TRP effector that has a diverse array of interactions with host proteins and also acts as a molecular mimic to promote infection and intracellular survival (Byerly et al., 2021). Altogether, these investigations indicate that *Ehrlichia* spp. have evolved complex mechanisms that act in a context-dependent manner in part through T1SS TRP effectors to promote infection and intracellular survival.

Studies of *E. chaffeensis* proteins, including TRP120, have defined multi-functional effectors that play important roles in both extracellular and intracellular contexts. Most recently, TRP120 has been defined as the first ligand mimic reported to activate multiple evolutionarily conserved signaling pathways including Notch, Wnt, and Hedgehog (Hh). As such, *E. chaffeensis* TRP120 has become a model to study how effectors interface with the host cell and exploit various cellular pathways and molecular processes to promote intracellular survival (Byerly et al., 2021). This review will provide a comprehensive overview of the multiple roles TRP120 plays during infection and highlight the recently defined examples of SLiM mimicry (SLiM-icry).

### TRP120 effector functions

**Figure 1:**
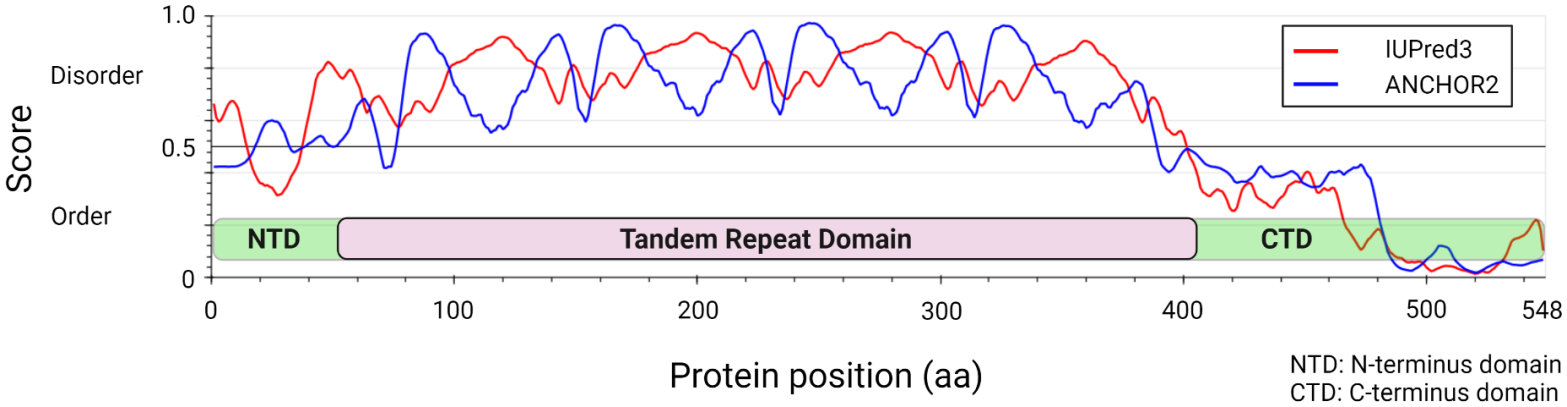
*Ehrlichia chaffeensis* TRP120 effector structural and disorder prediction. *In silico* analysis of *E. chaffeensis* TRP120 by disordered protein prediction reveals the unstructured region of the tandem repeat domain. The red line indicates disordered protein regions (IUPred3) (Erdős et al., 2021), and the blue line represents disordered protein binding domains (ANCHOR2) (Dosztányi et al., 2009; Mészáros et al., 2018).

Studies over the last decade have molecularly defined the *E. chaffeensis* TRP120 effector as a moonlighting protein that has multiple important functions during infection (Byerly et al., 2021). Structurally, TRP120 consists of a small N-terminus domain (51 aa) of unknown function, a central intrinsically disordered tandem repeat (TR) region (354 aa) consisting of four nearly identical 80 aa TRs, flanked by partial repeat sequences, and a C-terminal domain (142 aa) that harbors a type I secretion signal sequence (~50 amino acids) responsible for secretion through the T1SS (**Figure 1**) (Luo et al., 2011; Wakeel et al., 2011; Klema et al., 2018). TRP120 decorates the surface of infectious dense-cored ehrlichiae likely through a recently described mechanism in which secretion stalls before completion, creating a pseudoperiplasmic intermediate that is surface-exposed (Popov et al., 2000). The surface localization of TRP120 on infectious dense-cored ehrlichiae is known to contribute to host cell entry, and many studies have documented its intracellular role as a nucleomodulin with ability to bind host cell DNA and degrade host nuclear proteins (Popov et al., 2000; Zhu et al., 2011; Zhu et al., 2017).

**Figure 2:**
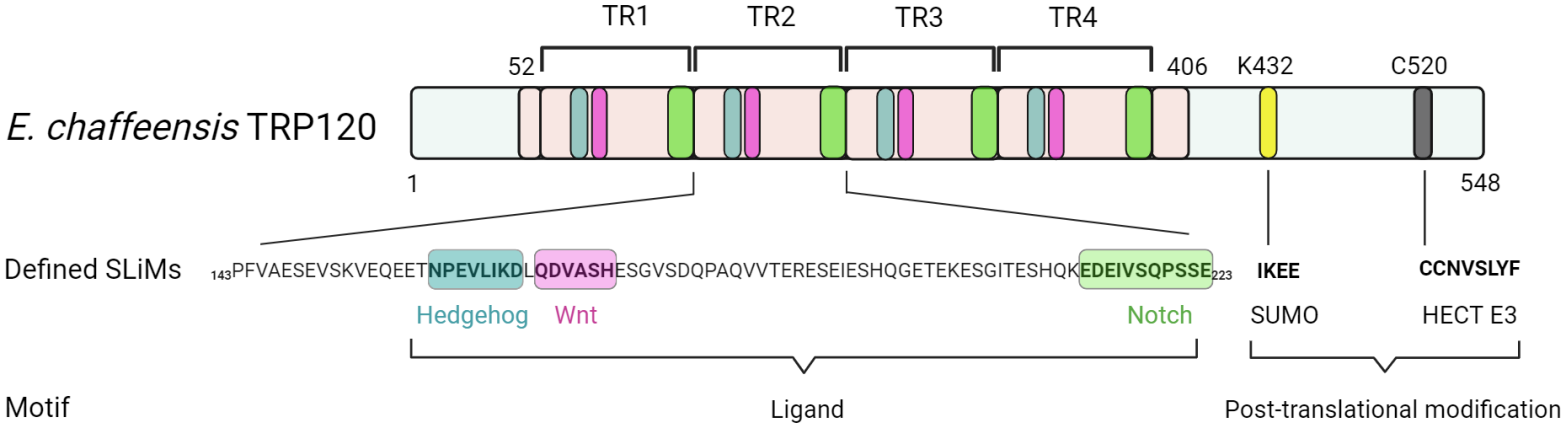
Functionally defined *E. chaffeensis* TRP120 effector SLiMs. Multiple SLiMs present in TRP120 have been identified and functionally characterized during infection. These include TRP120 Hedgehog, Wnt, and Notch SLiMs that function as receptor ligand mimetics as well as TRP120 SUMO and HECT E3 SLiMs involved in post-translational modifications.

Recent investigations have revealed that TRP120 has a canonical small ubiquitin-like modifier (SUMO) motif and a functional HECT E3 ligase catalytic domain in the C-terminal domain (**Figure 2**) (Dunphy et al., 2014; Zhu et al., 2017). Interestingly, distinct mechanisms by which *E. chaffeensis* TRP120 acquires or catalyzes PTMs through SUMOylation and HECT E3 ligase activity have been demonstrated to govern bacterial effector-host interactions or to dictate the fate of host nuclear proteins, respectively. Specifically, *E. chaffeensis* TRP120 is SUMOylated by the host machinery, which is critical for TRP120 effector-host interactions (Dunphy et al., 2014). The HECT E3 ligase domain of TRP120 has been associated with its functions as a nucleomodulin, mediating auto-ubiquitination as well as ubiquitination of multiple host substrates (Zhu et al., 2017; Mitra et al., 2018; Wang et al., 2020; Zhu and McBride, 2021). Moreover, TRP120 binds a GC-rich DNA motif that is similar to GC-rich motifs bound by eukaryotic transcription factors, suggesting a similar function in mediating host gene transcriptional regulation (Zhu et al., 2011). It is worth noting that two full-repeat sequences of the four consecutive TR domains of TRP120 are sufficient to bind a double-stranded DNA (dsDNA) and oligonucleotide probes containing GC-rich motifs (Zhu et al., 2011).

**Figure 3:**
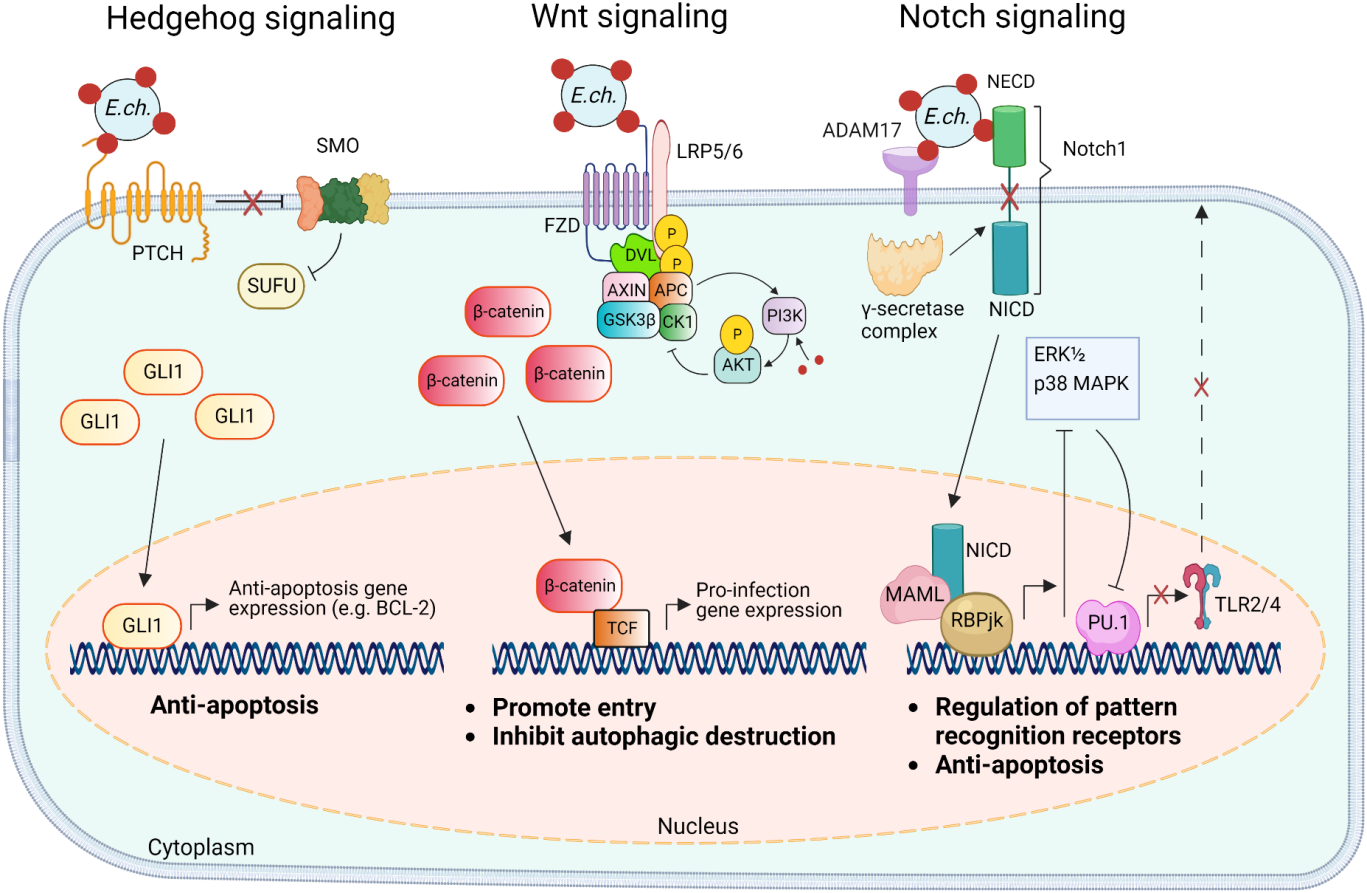
Model of *E. chaffeensis* TRP120 ligand SLiM activation of Hedgehog, Wnt, and Notch signaling pathways and downstream effects. The TRP120 Hedgehog ligand SLiM binds to the Hedgehog receptor (PTCH) to activate the GLI-1 zinc finger transcription factor. GLI1 translocation to the nucleus activates the transcription of the anti-apoptosis target genes such as BCL-2. In the canonical Wnt signaling pathway, the TRP120 Wnt ligand SLiM directly engages Fzd (Frizzled) 5 and recruits coreceptor LRP5/6 (lipoprotein receptor-related protein 5 and 6). Activation of Wnt signaling results in the disassembly of the β-catenin destruction complex [consisting of Axin, APC (adenomatous polyposis coli), GSK3β (glycogen synthase kinase 3 beta), and CK1 (casein kinase)], which allows accumulation of β-catenin in the cytoplasm and subsequent nuclear translocation and activation of Wnt target pro-infection genes and Wnt signaling promotes PI3K (phosphatidylinositol 3-kinase)/Akt signaling and downstream suppression of autophagy. The TRP120 Notch ligand SLiM interacts with ADAM17 (a disintegrin and metalloproteinase 17) and the Notch1 receptor resulting in receptor cleavage and nuclear translocation of NICD (Notch intracellular domain), the transcriptionally active form that interacts with Notch RBPjk (recombinant binding protein suppressor of hairless involved in Notch signaling) and MAML (mastermind-like protein 1) transcription factors proteins. This transcription complex activates transcription of Notch target genes, resulting in inhibition of ERK1/2 (extracellular signal-regulated kinases) and p38 MAPK (p38 mitogen-activated protein kinase) phosphorylation pathway and the downstream transcription factor PU.1 expression is repressed, inhibiting *TLR2/4* expression and apoptosis.

A rapidly expanding body of evidence has revealed that *E. chaffeensis* TRP120 harbors multiple short linear motifs (SLiMs) within the tandem repeat domain which serve as ligand mimetics that activate conserved cellular signaling pathways, including Notch, Wnt, and Hh (**Table 1**; **Figure 3**) (Rogan et al., 2021; Byerly et al., 2022; Patterson et al., 2022). Wnt signaling has been linked to cytoskeletal changes and phagocytosis that stimulate ehrlichial entry (Popov et al., 2000; Kumagai et al., 2010). Moreover, SLiM sequence-specific ligand-receptor interactions between TRP120 and Notch and Hh receptors also activate these pathways to promote ehrlichial infection (Rogan et al., 2021; Byerly et al., 2022; Patterson et al., 2022). These reports highlight the important role of TRP120 SLiM-icry in *Ehrlichia* pathobiology. Although functions are still unknown, a handful of eukaryotic-like SLiMs have also been predicted in other bacteria (Sámano-Sánchez and Gibson, 2020), suggesting a common mechanism through SLiM-mediated survival strategies employed by many bacterial pathogens.

**Table 1:** Ehrlichia chaffeensis TRP120 functionally defined short linear motifs (SLiMs).

SLiM sequence	Location	Eukaryotic ligand/conserved motif mimetic	Host target/receptor	Function/Outcome	Reference
Post-translational modification motif					
IKEE	C-terminus (431-434)	Canonical consensus SUMO motif (ψKxD/E, where ψ is a hydrophobic residue and “x” is any residue)	SUMO2/3 -TRP120-PCGF5	Enhance bacterial effector-host protein interactions	(Dunphy et al., 2014)
CCNVSLYF	C-terminus (520-527)	Canonical consensus HECT E3 ligase	PCGF5, ENO-1, FBW7	Autoubiquitination of TRP120 and ubiquitination of host cell substrates.	(Zhu et al., 2017; Mitra et al., 2018; Wang et al., 2020; Zhu et al., 2021)
Ligand motif					
QDVASH	TR^*^	Wnt (Wnt3a/5a)	Fzd2, 4, 5, 7, 9	Activation of Wnt signaling to promote infection and inhibit autolysosome generation and autophagic destruction	(Rogan et al., 2021)
EDEIVSQPSSE	TR	Notch (Jagged-1, DLL1, DLL4, TSP2)	Notch	Activation of canonical Notch signaling to downregulate TLR2/4 expression and promote intracellular survival	(Patterson et al., 2022)
NPEVLIKD	TR	Hedgehog (Hh)	PATCH2	Activation of Hh signaling to engage a BCL-2 anti-apoptotic cellular program that eventually promote infection	(Byerly et al., 2022)

*TR, tandem repeat domain.

### Short linear motifs

Since their discovery in the early 2000s, SLiMs are now recognized as protein interaction modules that play major roles in many protein interactions that govern cellular processes (Gouw et al., 2018). SLiMs are short linear amino acid sequences (3-10 aa) that are typically found within intrinsically disordered domains (Davey et al., 2012). Because of their linear and disordered nature, SLiMs have a high likelihood of arising *in nihilo*, and allow for high functional density in a given length of peptide (Davey et al., 2012; Van Roey et al., 2014). Despite lacking an inherent structure, SLiMs perform a variety of roles, challenging outdated models that protein function is tied to a defined tertiary conformation (Dunker et al., 2001). In general, SLiMs may be functionally divided into two categories: ligand motifs and PTM motifs. Ligand motifs facilitate non-catalytic protein interactions for purposes including complex formation and enzyme recruitment, while PTM motifs enable catalytic alteration of specific sites (Van Roey et al., 2014; Kumar et al., 2022). There are over 100,000 SLiMs predicted in the human proteome that perform a myriad of functions. Some of these SLiMs have evolved in pathogens to mimic eukaryotic functions (Tompa et al., 2014; Sámano-Sánchez and Gibson, 2020).

Pathogen SLiM-icry is a relatively new concept but is not uncommon. In fact, SLiMs have been identified in secreted effector proteins in a variety of bacterial pathogens (reviewed in (Sámano-Sánchez and Gibson, 2020)). SLiM-mediated virulence mechanisms and associated pathogens include, but are not limited to, tyrosine phosphorylation by *Anaplasma phagocytophilum*, actin remodeling by *Listeria monocytogenes*, and adaptor protein recruitment by *Mycobacterium tuberculosis* (Sámano-Sánchez and Gibson, 2020). While all these bacteria utilize SLiM-icry during infection, a growing body of evidence illustrates that *E. chaffeensis* is especially adept at utilizing this motif-driven infection strategy. In particular, TRP120 has emerged as the master manipulator of cell signaling utilizing multiple distinct SLiMs to mimic sequences found in human Notch, Wnt, and Hh ligands (**Table 1**). TRP120 interacts directly with respective receptors to activate host cell signaling (**Figure 3**) (Rogan et al., 2021; Byerly et al., 2022; Patterson et al., 2022). Moreover, there are numerous PTM SLiMs in TRP120 (Dunphy et al., 2014; Rogan et al., 2021; Byerly et al., 2022; Patterson et al., 2022). Ligand motifs have long been recognized as mediators of protein-protein interactions, but SLiMs that initiate signaling pathways *via* direct receptor activation were unknown until discovered in TRP120.

*E. chaffeensis* TRP120 is the only bacterial effector known to act as a ligand mimic and possess multiple SLiMs that directly activate distinct signaling pathways. As such, TRP120 represents the first example of what may be a more ubiquitous type of bacterial effector protein with such capability. Usurpers of cell signaling can provide enormous insight to SLiM functionality, role in disease pathology, potential drug and vaccine targeting, and fundamental understanding of molecular ligand-receptor interactions resulting in biosignaling. Developing *E. chaffeensis* TRP120 as a model for investigating SLiM-icry will accelerate our understanding of the role SLiMs play in interkingdom interactions leading to the development of effective countermeasures and therapeutics for many diseases.

## Post-translational modification SLiMs

### TRP120 SUMOylation mediates effector-host interactions

While some SLiMs may serve as sites for structural modification or proteolytic cleavage, perhaps the most well-known SLiMs are those that function as recognition sites for the addition of modifications such as phosphate and ubiquitin. As these modifications can dramatically change protein function and interactions, pathogens also utilize PTMs to interface with the cell and function in the eukaryotic environment (Sámano-Sánchez and Gibson, 2020; Kumar et al., 2022). One such TRP120 SLiM allows for the conjugation of SUMO, which influences *Ehrlichia*-host interactions (Dunphy et al., 2014; Mitra et al., 2018). SUMOylation of *E. chaffeensis* TRP120 with SUMO2/3 occurs in the C-terminal at K432, part of a known SLiM that serves as a SUMOylation site. The full motif sequence (IKEE) found in TRP120 consists of only four residues and is consistent with the canonical SUMOylation consensus sequence (Φ(K)xD/E), where Φ denotes a hydrophobic amino acid and x denotes any amino acid (**Table 1**, **Figure 2**) (Rodriguez et al., 2001; Dunphy et al., 2014; Kumar et al., 2022). This particular SLiM is curated in the ELM database and is also found in many SUMOylated proteins such as human glutamate receptor interacting protein 1, progesterone receptor, and Sp3 transcription factor (Abdel-Hafiz et al., 2002; Kotaja et al., 2002; Sapetschnig et al., 2002; Kumar et al., 2022). The canonical SUMOylation SLiM is the sole SUMO conjugation site in TRP120 as alanine substitution of K432 or E434 both abolish TRP120 SUMOylation. Furthermore, abrogation of TRP120 SUMOylation *via* alanine substitution significantly hinders association with SUMO-interacting motif (SIM)-containing host interaction partners, γ-actin, myosin-X, and Golgi-localizing, γ-adaptin ear domain homology, and the ADP ribosylation factor (ARF)-binding proteins (Luo et al., 2011; Dunphy et al., 2014).

**Figure 4:**
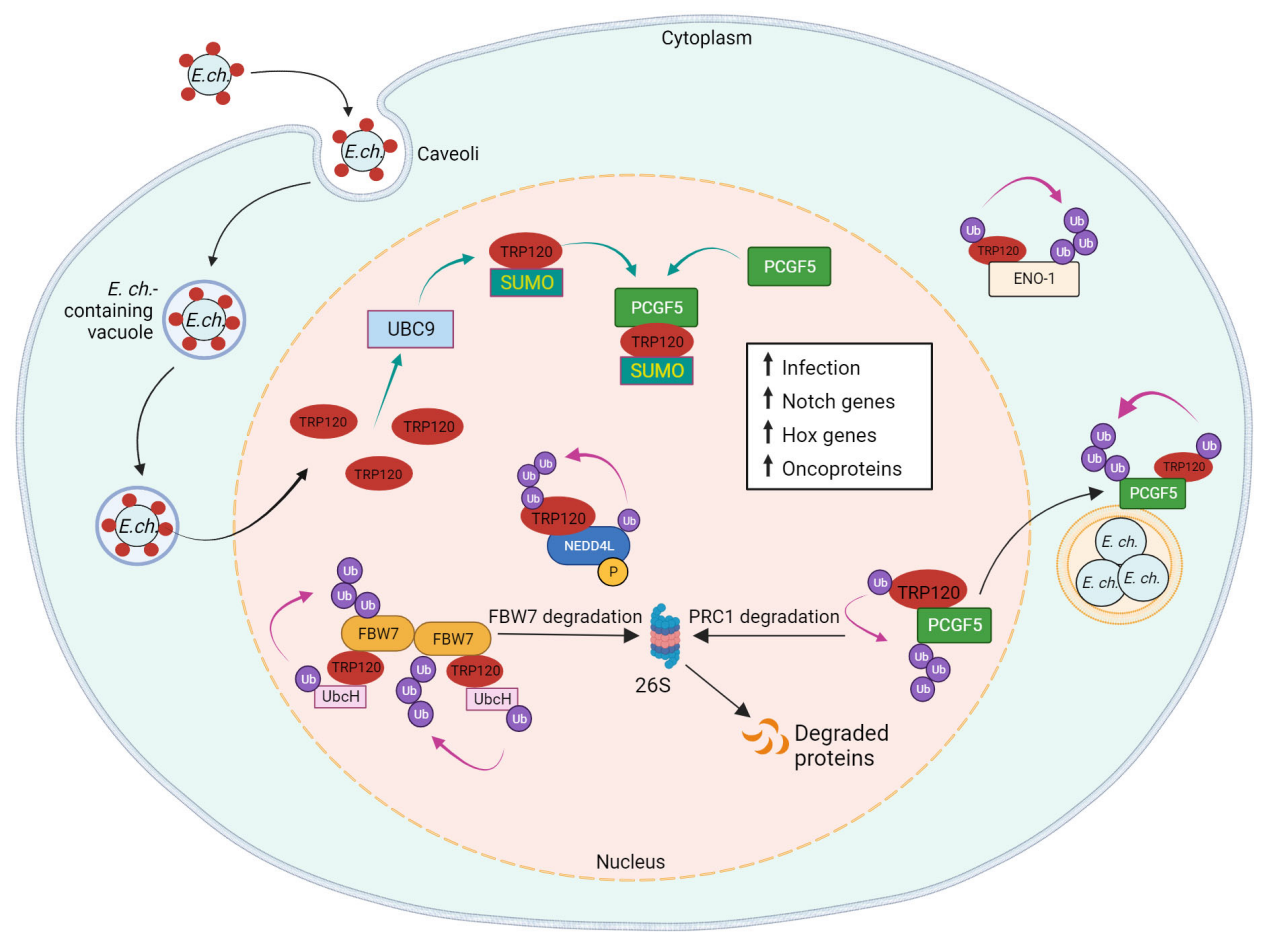
Model of *E. chaffeensis* TRP120 effector E3 ubiquitin ligase activity and PTM SLiMs that mediate effector-host interactions. During infection, TRP120 is SUMOylated at canonical SUMO motif by host cell PTM machinery UBC9 (ubiquitin conjugating enzyme 9), which promotes direct interaction of TRP120 with PCGF5 (polycomb group RING finger protein 5). Possessing intrinsic HECT E3 Ub ligase activity, TRP120 physically interacts with host NEDD4L (neural precursor cell expressed developmentally downregulated gene 4-like) to mediate self and substrate ubiquitination. Within the host nucleus, TRP120 utilizes its HECT E3 Ub ligase activity to target multiple host cell substrates, including FBW7 (F-box and WD repeat domain-containing 7), PCGF5 and ENO-1 (alpha-enolase 1) for Ub-mediated degradation. TRP120 binds to Notch negative regulator, FBW7, and ubiquitinates FBW7 with K48-Ub chains, resulting in the proteasomal degradation of FBW7 and upregulation of Notch genes and oncoproteins involved in cell survival. SUMOylated TRP120 binds and ubiquitinates PCGF5 resulting in PCGFs degradation and the upregulation of Hox genes. TRP120-mediated ENO-1 ubiquitination and subsequent degradation disrupts the glycolytic flux and promotes infection.

SUMOylation machinery is exclusive to eukaryotes, but bacteria and viruses are known to modulate host SUMOylation machinery during infection (Ribet and Cossart, 2010; Srikanth and Verma, 2017; Fan et al., 2022). Pathogens disrupt SUMOylation through various mechanisms such as the degradation of host SUMOylation machinery by *L. monocytogenes* or the mimicry of SUMO deconjugation proteins by *Xanthomonas campestris* (Hotson et al., 2003; Ribet et al., 2010; Hamon et al., 2012). However, while numerous viral proteins are known SUMO substrates, few SUMO substrates have been described in bacteria (Ribet and Cossart, 2010; Srikanth and Verma, 2017; Fan et al., 2022). Notably, TRP120 is the first of only three SUMOylated bacterial effectors ever reported, the others being APH1387 (AmpA) and APH0032 of *A. phagocytophilum* (Dunphy et al., 2014; Beyer et al., 2015; Oki et al., 2016). As the interactome of TRP120 includes many eukaryotic proteins containing SIMs, SUMOylation of TRP120 is responsible for facilitating interactions with host proteins that are crucial for bacterial survival (**Figure 4**) (Luo et al., 2011; Dunphy et al., 2014; Mitra et al., 2018).

SUMOylation can also influence protein-protein interactions independent of SIMs. Polycomb group ring finger protein 5 (PCGF5), a strong interaction partner of TRP120, interacts with the tandem repeat domain of TRP120, but does not possess a SIM. Nevertheless, the interaction between PCGF5 and TRP120 is significantly impaired after the disruption of TRP120 SUMOylation (Luo et al., 2011; Dunphy et al., 2014). PCGF5 and other isoforms are components of the polycomb repressive complex (PRC) which negatively regulates gene expression through histone modifications (Aranda et al., 2015). The degradation of PCGF5 *via* TRP120-facilitated ubiquitination during *E. chaffeensis* infection results in the increased expression of Hox genes that are regulated by PRC (Zhu et al., 2017).

The importance of TRP120 SUMOylation by host cell machinery is further illustrated by its influence on ehrlichial infection. A pretreatment with anacardic acid, an inhibitor of SUMOylation machinery, significantly decreases bacterial load in addition to TRP120/PCGF5 colocalization and interaction. Significant decreases in ehrlichial inclusions per cell were also observed in anacardic acid-treated cells compared to vehicle control (Dunphy et al., 2014). Therefore, the mimicry of a eukaryotic SUMOylation SLiM by TRP120 plays a significant role in effector-host interactions during infection.

## SLiM-mediated ligand mimicry and evasion of innate defenses

### TRP120 Notch SLiM ligand mimicry and regulation of pattern recognition receptors

The Notch signaling pathway is classically defined as an evolutionarily conserved regulator of cell-fate and is particularly active during early development and tissue homeostasis (Siebel and Lendahl, 2017). Notch signaling also plays important roles in major histocompatibility complex (MHC) class II expansion, B and T cell differentiation, and innate immune mechanisms that involve autophagy and apoptosis (Palaga et al., 2013). Activation and modulation of Notch signaling by bacterial pathogens (e.g., *Salmonella typhimurium, Mycobacterium bovis, Bacillus anthracis, Pseudomonas aeruginosa*, and *Staphylococcus aureus*) influence cellular changes such as the upregulation of A disintegrin and metalloprotease 17 (ADAM17), that cleaves the Notch receptor, and upregulation of suppression of cytokine signaling 3 (SOCS3), a negative regulator of numerous cytokines and toll-like receptors (TLRs) (Gómez et al., 2004; Gómez et al., 2005; Narayana and Balaji, 2008; Larabee and Ballard, 2014). Yeast two hybrid (Y2H) analysis revealed that TRP120 interacts with proteins involved in activating and regulating Notch signaling, including ADAM17 and F-BOX and WD repeat domain-containing 7 (FBW7) (Luo et al., 2011). Canonical Notch activation occurs in response to Notch ligands (Delta-like ligand (DLL)1/-3/-4 and Jagged 1/-2) engaging the Notch extracellular domain (NECD) expressed on neighboring cells followed by receptor cleavage by ADAM17 and γ-secretase (Lina et al., 2016; Patterson et al., 2022). The cleaved Notch intracellular domain (NICD) translocates to the nucleus where it binds transcriptional co-activators, recombination binding protein suppressor of hairless (RBPjK) and mastermind-like protein (MAML), resulting in Notch gene transcription. A recent investigation demonstrated that TRP120 activates Notch signaling *via* SLiM-icry. Studies have shown that an 11 amino acid peptide (EDEIVSQPSSE) located in the TR domain of TRP120 can activate Notch and upregulate Notch target gene expression (**Table 1**). Notably, as a result of Notch activation, the expression of classical pattern recognition receptors (PRRs) and TLR2/4 is downregulated. Mechanistically, TRP120-mediated Notch activation results in inhibition of the extracellular signal regulated kinase (ERK) 1/2 and p38 mitogen-activated protein kinase (MAPK), which are required for expression of PU.1, an essential transcription factor that regulates expression of TLR2/4 (**Figure 3**) (Lina et al., 2016).

### TRP120 Notch SLiM-icry and apoptosis regulation

Apoptosis is an innate defense mechanism activated to eliminate intracellular pathogens (Sly et al., 2003), but *E. chaffeensis* has evolved a multifaceted strategy to inhibit apoptosis to prolong host cell survival. The process of programmed cell death (apoptosis) is usually engaged during early stages of infection, but *E. chaffeensis* successfully regulates host cell apoptosis, in part, by upregulating anti-apoptotic proteins such as myeloid cell leukemia sequence 1 protein (MCL-1), immediate early response 3 (IER3), baculoviral IAP repeat containing 3 (BirC3), and B-cell lymphoma 2 (BCL-2), while simultaneously downregulating inducers of apoptosis such as hematopoietic cell kinase (HCK), Bcl-2-interacting killer (BIK), and BCL2/Adenovirus E1B 19-Kd protein-interacting protein 3 (BNIP3L) (Zhang et al., 2004; Luo et al., 2011). In addition to decreasing TLR2/4 expression to avoid recognition, *E. chaffeensis* TRP120 activates Notch signaling and degrades the nuclear tumor suppressor FBW7, which is a negative regulator of major oncoproteins such as NICD, MCL-1, c-Jun, and cMYC. Through TRP120 HECT E3 Ub ligase activity, FBW7 is ubiquitinated leading to proteasomal degradation, which stabilizes NICD and other oncoproteins (**Figure 4**) (Wang et al., 2020). This finding supports previous data which demonstrated that siRNA knockdown of FBW7 results in the enhancement of infection, indicating that the TRP120-FBW7 interaction is associated with *E. chaffeensis* survival (Wang et al., 2020). Interestingly, *E. chaffeensis* has evolved a multifaceted utilization of Notch signaling. *Ehrlichia-*induced Notch activation has recently been linked to stabilization of the X-linked inhibitor of apoptosis (XIAP), which prevents intrinsic apoptosis during infection (Patterson et al., 2023). The SLiM-mediated activation and repurposing of Notch signaling demonstrates the power of SLiM-icry for intracellular pathogen survival.

### TRP120 Wnt SLiM-icry and ehrlichial entry

The Wnt signaling pathway is an evolutionarily conserved eukaryotic signaling cascade that not only regulates cell fate, development, and cell polarity, but also mediates innate immunity-associated events, including autophagy, cytokine expression, and phagocytosis (Schaale et al., 2011; Kim et al., 2013; Petherick et al., 2013). Canonical Wnt signaling is the most well-studied Wnt pathway that is also referred to as the β-catenin-dependent signaling pathway (Nusse and Clevers, 2017). Typically, the activation of the pathway is triggered through interactions between Wnt ligands and Frizzled (Fzd) receptors and the canonical pathway co-receptor lipoprotein receptor-related protein 5/6 (LRP5/6). The initial signal is then transduced to the intracellular mediator Disheveled (Dvl) followed by the recruitment of the β-catenin destruction complex consisting of Axin, adenomatous polyposis coli (APC), glycogen synthase kinase 3β (GSK3β), and casein kinase 1 (CK1) to the Fzd-Dvl complex at the plasma membrane, therefore freeing β-catenin from degradation. This leads to the accumulation of β-catenin in the cytoplasmic pool and translocation into the nucleus where it binds T-cell factor (TCF), at the Wnt response element (WRE) and activates transcriptional expression of the Wnt target genes (Nusse and Clevers, 2017).

Although dysregulation of the pathway has been implicated in multiple cancer types, growing investigations indicate that bacterial pathogens manipulate Wnt signaling through numerous secreted effectors to enhance infection (Rogan et al., 2019). Moreover, siRNA inhibition of the Wnt signaling pathway blocks the uptake of microspheres coated with recombinant TRP120 (Luo et al., 2016), supporting previous studies that suggested TRP120 functions as an *E. chaffeensis* adhesin that stimulates bacterial entry by activating Wnt signaling. Moreover, siRNA silencing of Wnt signaling components, including β-catenin, CK1, Fzd5, Fzd9, and LRP6, significantly decreases *E. chaffeensis* infection, while silencing Wnt antagonist Dickkopf-related protein 3 (DKK3) promotes infection (Luo et al., 2016). Notably, other bacteria such as *S. enterica, M. tuberculosis, Clostridium difficile, P. aeruginosa*, and *Escherichia coli* also exploit the Wnt signaling pathway (Rogan et al., 2019), implicating evolutionarily conserved Wnt signaling as an important pathway for pathogen manipulation and infection.

*E. chaffeensis* TRP120 plays a major role in directly manipulating Wnt signaling that ultimately promotes infection. The interactions of TRP120 with various components of the Wnt signaling pathway, including positive regulators such as protein phosphatase 3 regulatory subunit B alpha (PPP3R1) and vacuolar protein sorting protein 29 (VPS29), and negative regulators such as AT-rich interactive domain-containing protein 1B (ARID1B), centrosomal protein 164 (CEP164), Kelch-like protein 12 (KLHL12), interleukin enhancer-binding factor 3 (ILF3), and LIM domain only 2 (LMO2), were identified by using a Y2H system (Luo et al., 2011). More importantly, a recent investigation has ascertained the molecular mechanism by which *E. chaffeensis* TRP120 effector utilizes sequence-specific Wnt SLiM-icry to activate Wnt signaling in human monocytes (Rogan et al., 2021). Specifically, strong colocalization between *E. chaffeensis* and Fzd2, 4, 5, 7, and 9 was confirmed by the direct binding between TRP120 and these Fzd receptors. A 6 amino acid SLiM (QDVASH) was identified in TRP120 that is homologous to Wnt ligands. Further investigation using mutant SLiM peptides and an α-TRP120-Wnt-SLiM antibody demonstrated that the TRP120 Wnt SLiM activates the canonical Wnt pathway and promotes *E. chaffeensis* infection (Rogan et al., 2021). Notably, an antibody specific to the Wnt SLiM inhibited both recombinant TRP120 and *E. chaffeensis* activation of Wnt signaling. These findings revealed the molecular basis of Wnt activation by *E. chaffeensis* and identified a previously undefined Wnt SLiM that has broader implications for infection as well as our general understanding of Wnt ligand-receptor biology.

### TRP120 Wnt SLiM-icry regulation of autophagic destruction

Autophagy is an evolutionarily conserved pathway that regulates lysosomal degradation of intracellular components (Mizushima, 2018; Levine and Kroemer, 2019). Although this process is activated during stress conditions to maintain cellular homeostasis by removing misfolded or aggregated proteins, and clearing damaged organelles, such as mitochondria, endoplasmic reticulum and peroxisomes (Glick et al., 2010), autophagy plays a key role in innate immune defense against microbial infection by directly targeting these invaders for autophagic destruction in the lysosomes, thereby preventing intracellular infection (Deretic et al., 2013; Tominello et al., 2019).

The autophagic process is characterized by the regulated induction and formation of the double membrane autophagosomes in the cytoplasm. Phagophores recognize and encapsulate tagged cytoplasmic components or intracellular pathogens for cellular homeostasis or host defense purposes. Phagophores mature into autophagosomes which then fuse with lysosomes to form single-membrane autolysosomes. The autolysosomes degrade autophagic cargos to recycle proteins and generate cellular energy (Li et al., 2019; Nakatogawa, 2020). The major signaling protein mediating the autophagic process is mechanistic target of rapamycin (mTOR) kinase which is tightly regulated by several signal transduction pathways including the Wnt and phosphoinositide 3-kinase (PI3K)/ATP dependent tyrosine kinase (Akt) signaling pathways (Yuan et al., 2013; Ma et al., 2017). Specifically, mTOR is activated downstream of Akt and PI3K kinases to inhibit autophagy (Manning and Cantley, 2007). Glycogen synthase kinase-3 (GSK3) inhibits the mTOR pathway by phosphorylating tuberous sclerosis complex 2 (TSC2) in a manner dependent on AMPK-priming phosphorylation (Inoki et al., 2006). Further, the Akt-mediated GSK3 phosphorylation depends on activation of Wnt signaling (Ma et al., 2011). Therefore, Wnt signaling appears to play an essential role in the inhibition of autophagy by regulating activation of the mTOR pathway (Petherick et al., 2013; Fu et al., 2014).

Indeed, an investigation demonstrated that *E. chaffeensis* TRP120-mediated activation of Wnt-PI3K-mTOR signaling inhibits autolysosome generation and autophagic destruction to establish a favorable niche for intracellular replication (Lina et al., 2017). Either *E. chaffeensis* infection or treatment with recombinant TRP120 protein activates Wnt and PI3K/Akt pathways as well as mTOR signaling and regulation of nuclear translocation of transcription factor EB (TEFB), inhibiting lysosomal biogenesis and autolysosomal fusion with *E. chaffeensis*-containing vacuole (Lina et al., 2017). During *E. chaffeensis* infection, phosphorylation of PI3K and Akt is increased, while phosphatase and tensin homolog (PTEN), a PI3K/Akt pathway inhibitor, decreases over the course of infection, implicating an essential role for PI3K/Akt pathway activation during infection (Lina et al., 2017). Further, siRNA knockdown of Rheb, a GTPase that activates mTOR, confirmed a role for mTOR signaling during infection. Knockdown of both Rheb and phospho-p70 S6 kinase decreased *E. chaffeensis* infection (Lina et al., 2017). Thus, the activation of Wnt, PI3K/Akt and mTOR signaling is required for *E. chaffeensis* survival.

Wnt signaling regulates the PI3K/Akt pathway *via* GSK3. Specifically, GSK3, a negative regulator of Wnt/β-catenin, is a critical downstream element of the PI3K/Akt pathway and regulates mTOR by inducing TSC2, an mTOR negative regulator (Inoki et al., 2002; Inoki et al., 2006; Zhang et al., 2006; Vigneron et al., 2011). During *E. chaffeensis* infection, an increased level of phosphorylated GSK3 was detected which is abrogated following the treatment with a specific Wnt-Dvl inhibitor, while the level of TSC2 was decreased (Lina et al., 2017). Additionally, treatment with either an Akt inhibitor or GSK3 inducer resulted in a significant decrease of *E. chaffeensis*-infected cells. Notably, an increased level of phosphorylated GSK3 was detected in cells stimulated with recombinant TRP120 (Lina et al., 2017), suggesting that *E. chaffeensis* TRP120 simultaneously activates the PI3K/Akt pathway while inactivating GSK3 to inhibit TSC2. Therefore, GSK3 serves to integrate PI3K/Akt and Wnt signals in the induction of the mTOR pathway during *E. chaffeensis* infection. Further, TSC2 inhibition leads to activation of mTORC1 and subsequent phosphorylation and inhibition of the nuclear translocation of TFEB, a transcription factor that coordinates expression of lysosomal hydrolases, membrane proteins and genes involved in autophagy signaling. Indeed, the localization of TFEB was observed in the cytoplasm during *E. chaffeensis* infection and was modulated by *E. chaffeensis*-mediated Wnt activation (Lina et al., 2017). These findings indicate that *E. chaffeensis* exploits Wnt-PI3K-mTOR signaling in part to regulate mTOR signaling and TFEB nuclear localization, thereby inhibiting autolysosomal generation and subsequent autophagic destruction to survive intracellularly.

### TRP120 Hh SLiM-icry and apoptosis regulation

The Hh signaling pathway plays vital roles in embryogenesis as well as cell differentiation, proliferation, and survival and has been extensively studied in the context of developmental biology (Ingham and McMahon, 2001; Jia et al., 2015). The Hh signaling pathway is initiated by the binding of hedgehog family ligands (Sonic hedgehog, Indian hedgehog, or Desert hedgehog) to the Patched receptor (PTCH). In the absence of Hh ligand, PTCH continually represses the Smoothened (SMO) protein; however, this activity is disabled upon Hh ligand-binding, enabling SMO to activate members of glioma-associated oncogene (GLI) family of transcription factors (GLI-1, GLI-2, and GLI-3). This conserved pathway culminates in the regulation of the genes controlling various cellular processes including immune response, autophagy, and apoptosis (Taipale et al., 2002; Lee et al., 2016; Lan et al., 2017; Smelkinson, 2017).

Because the Hh pathway regulates aspects of innate immunity and cell survival, both viral and bacterial pathogens exploit Hh signaling during infection. The Hh pathway is activated by Epstein-Barr virus and *Helicobacter pylori*, amongst other pathogens, but the precise mechanisms and purposes behind this activity are unclear (Smelkinson, 2017). *E. chaffeensis* has emerged as one of the best understood modulators of Hh signaling as it uses SLiM-icry to activate the Hh pathway and inhibit apoptosis. Protein alignments revealed that a short repeated sequence in the TRP120 TR domain exhibits homology with Hh ligands (Byerly et al., 2022). The identified Hh SLiM (NPEVLIKD) was consistent in size and location (TR domain) with that of other SLiMs (Wnt and Notch) involved in SLiM-icry (Rogan et al., 2021; Byerly et al., 2022; Patterson et al., 2022).

Informational spectrum method (ISM) also predicted a similar function between Desert and Indian Hh ligands and the TRP120 TR domain containing the Hh SLiM. TRP120 was shown to interact with PTCH2 *via* colocalization and co-immunoprecipitation studies. Importantly, this interaction was also found to activate the PTCH2 receptor, as treatment of THP-1 cells with recombinant TRP120 elicited GLI-1 activation and Hh gene expression consistent with recombinant Sonic Hh ligand. Additionally, inhibiting TRP120 Hh SLiM-icry by antibody or mutating with alanine/glycine substitutions prevents GLI-1 activation, confirming that TRP120 Hh SLiM (NPEVLIKD) mediates activation of Hh signaling (**Table 1**). Therefore, *E. chaffeensis* activates Hh, Notch, and Wnt signaling during infection *via* SLiM-icry (Byerly et al., 2022).

This unique cellular signaling reprogramming mechanism is particularly important for ehrlichial infection. Knocking down Hh pathway members such as PTCH2, SMO, and GLI-1 all significantly decreased *E. chaffeensis* infection. The decreased ehrlichial burden was attributed to the absence of an anti-apoptotic profile typically induced by Hh pathway activation during infection. *E. chaffeensis* was shown to increase expression of the anti-apoptotic protein, BCL-2, in THP-1 cells, which countered etoposide-induced apoptosis. However, ehrlichial infection in the presence of a Hh signaling inhibitor, vismodegib, resulted in significantly lower levels of BCL-2 and increased caspase activation and apoptosis (Byerly et al., 2022).

SLiM-mediated mimicry of Hh ligand is a powerful and unique bacterial survival strategy. Multiple TRP120 SLiMs converge on signaling pathways that inhibit apoptosis and other host cell defense mechanisms. Through SLiM-icry, TRP120 effectively usurps normal cellular signaling processes to induce a cellular environment that is beneficial for the replication and persistence of *E. chaffeensis* in mononuclear phagocytes.

## Conclusions and future directions

Through coevolution with the host cell, obligately intracellular bacterial pathogens have evolved a variety of mechanisms to evade detection by innate host defenses. Molecular mimicry of eukaryotic ligands is a novel mechanism that enables *Ehrlichia* to subvert innate immune defenses to promote intracellular survival. An array of TRP120 SLiMs engage distinct host receptors to simultaneously activate and regulate conserved cellular signaling pathways, including Hedgehog, Wnt, and Notch. Moreover, the contributions of SLiMs that are involved in post-translational modifications such as SUMOylation also contribute to this molecular strategy of cellular exploitation. TRP120 SLiM-icry as a survival strategy to repurpose eukaryotic pathways to benefit infection by *E. chaffeensis* highlights the importance of this mechanism in pathobiology. Thus, defining these molecular motifs and cellular interactions during infection will likely lead to the development of novel antimicrobials and therapeutics.

**Figure 5:**
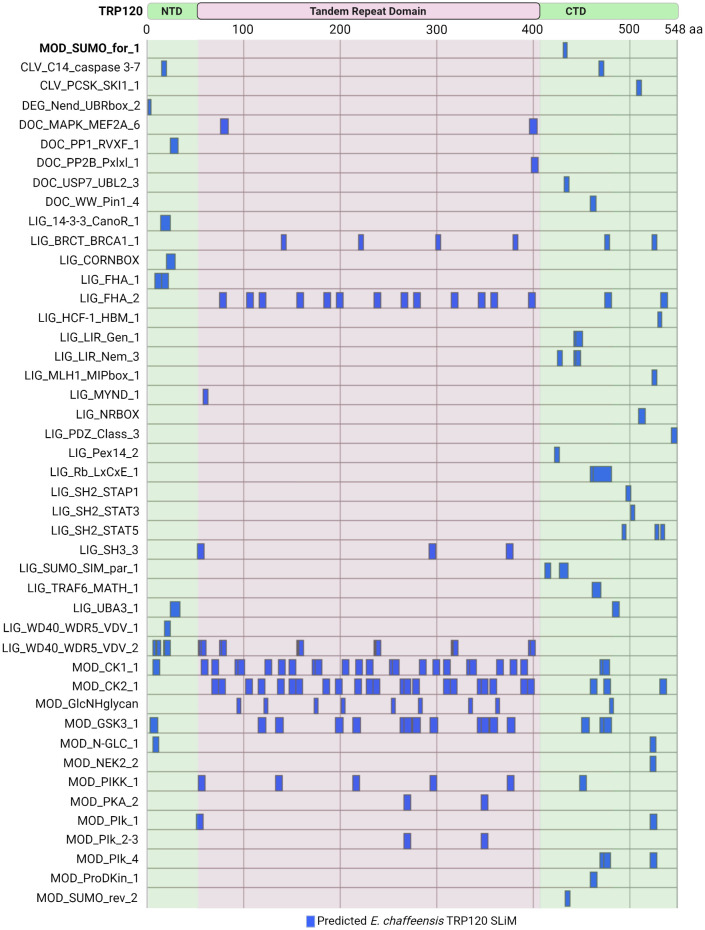
*Ehrlichia chaffeensis* TRP120 effector SLiMs identified by the ELM resource. *In silico* analysis by the Eukaryotic Linear Motif (ELM) resource (http://elm.eu.org/) reveals over 45 unique SLiM classes for a total of 184 SLiM instances in TRP120. Experimentally verified SliMs include MOD_SUMO_for_1 (bolded). Most of the predicted SLiMs are classified into either Ligand (LIG) or Modification (MOD) classes, while Docking (DOC), Cleavage (CLV), and Degradation (DEG) motifs are also represented.

Current knowledge regarding TRP120 SLiM-icry only scratches the surface of a broader and far more complex area of study. The Eukaryotic Linear Motif (ELM) resource annotates and detects SLiMs by providing both a repository of annotated, experimentally validated SLiM data and an open access database which can be used to identify SLiMs in protein sequences (Kumar et al., 2022). Of the 3,953 experimentally validated SLiMs curated in the ELM database, 45 unique SLiMs and 184 instances are identified in TRP120 (**Figure 5**). For example, the experimentally validated TRP120 SUMO motif was identified by the ELM database (MOD_SUMO_for_1). However, there are newly discovered SLiMs that have not been curated in the ELM database such as the Wnt, Notch, and Hh SLiM ligands, suggesting that there are numerous SLiMs remaining to be discovered and functionally characterized.

Many of these ELM predicted TRP120 SLiMs are phosphorylation sites for kinases including MAPK family members (DOC_MAPK_MEF2A_6), Src Homology 2 (SH2) family members (LIG_SH2_STAP1, LIG_SH2_STAT3, and LIG_SH2_STAT5), GSK3 (MOD_GSK3_1), calcineurin kinases (DOC_PP2B_Pxlxl_1), and casein kinase (MOD_CK1_1 and MOD_CK2_1). Other notable predicted SLiM candidates include phosphatase docking motifs (DOC_PP1_RVXF_1) and SLiMs that mediate interactions with tumor suppressor proteins such as breast cancer type 1 susceptibility protein (LIG_BRCT_BRCA1_1) and retinoblastoma protein (LIG_Rb_LxCxE_1), as well as other proteins important in the regulation of cell growth and division (LIG_HCF-1_HBM_1). Additionally, functional SLiMs described in other *Ehrlichia* TRP effectors, such as a MYND-binding domain integral in the nuclear translocation of TRP47 (Kibler et al., 2018) are also predicted in TRP120, but there are many more that need to be experimentally validated.

The vast array of validated and predicted SLiMs in TRP120 illustrates their capacity to impart a significant amount of control over cellular signaling to a single effector. As demonstrated by TRP120, this phenomenon has tremendous cellular reprogramming potential and must be understood to develop countermeasures for intracellular pathogens. Further work is necessary to fully characterize the predicted SLiMs in TRP120, identify novel SLiMs, and map out the complex effects that TRP120 has on cellular signaling pathways and beyond. The knowledge acquired from using TRP120 as a model for study will inevitably lead to the discovery of other artifice effectors that extend our understanding of SLiM-icry by pathogens and provide insight into the complex and multifaceted roles of TRP120 that have yet to be explored.

## Author contributions

NAP, RNS, and D-CB drafted the manuscript. JWM organized, directed, and contributed to the writing and editing of the manuscript. All authors have read and approved the submitted version. All authors contributed to the article and approved the submitted version.
